# Introducing novel and comprehensive models for predicting recurrence in breast cancer using the group LASSO approach: are estimates of early and late recurrence different?

**DOI:** 10.1186/s12957-018-1489-0

**Published:** 2018-09-12

**Authors:** Majid Akrami, Peyman Arasteh, Tannaz Eghbali, Hadi Raeisi Shahraki, Sedigheh Tahmasebi, Vahid Zangouri, Abbas Rezaianzadeh, Abdolrasoul Talei

**Affiliations:** 1 Breast Diseases Research Center, Shiraz University of Medical Sciences, Shiraz, Iran; 2 Non-communicable Diseases Research Center, Fasa University of Medical Sciences, Fasa, Iran; 3 Department of Biostatistics and Epidemiology, Faculty of Health, Shahrekord University of Medical Sciences, Shahrekord, Iran; 4 Surgical Oncology Division, General Surgery Department, Shiraz University of Medical Sciences, Shiraz, Iran

**Keywords:** Breast cancer, Early, Late, Recurrence, Prediction, Model

## Abstract

**Background:**

In here, we constructed personalized models for predicting breast cancer (BC) recurrence according to timing of recurrence (as early and late recurrence).

**Methods:**

An efficient algorithm called group LASSO was used for simultaneous variable selection and risk factor prediction in a logistic regression model.

**Results:**

For recurrence < 5 years, age (OR 0.96, 95% CI = 0.95–0.97), number of pregnancies (OR 0.94, 95% CI = 0.89–0.99), family history of other cancers (OR 0.73, 95% CI = 0.60–0.89), hormone therapy (OR 0.76, 95% CI = 0.61–0.96), dissected lymph nodes (OR 0.98, 95% CI = 0.97–0.99), right-sided BC (OR 0.87, 95% CI = 0.77–0.99), diabetes (OR 0.77, 95% CI = 0.60–0.98), history of breast operations (OR 0.38, 95% CI = 0.17–0.88), smoking (OR 5.72, 95% CI = 2.11–15.55), history of breast disease (OR 3.32, 95% CI = 1.92–5.76), in situ component (OR 1.58, 95% CI = 1.35–1.84), tumor necrosis (OR 1.87, 95% CI = 1.57–2.22), sentinel lymph node biopsy (SLNB) (OR 2.90, 95% CI = 2.05–4.11) and SLNB+axillary node dissection (OR 3.50, 95% CI = 2.26–5.42), grade 3 (OR 1.79, 95% CI = 1.46–2.21), stage 2 (OR 2.71, 95% CI = 2.18–3.35), stages 3 and 4 (OR 5.01, 95% CI = 3.52–7.13), and mastectomy+radiotherapy (OR 2.97, 95% CI = 2.39–3.68) were predictors of recurrence < 5 years. Moreover, relative to mastectomy without radiotherapy (as reference for comparison), quadrantectomy without radiotherapy had a noticeably higher odds ratio compared to quadranectomy with radiotherapy for recurrence < 5 years. (OR 17.58, 95% CI = 6.70–46.10 vs. OR: 2.50, 95% CI = 2–3.12).

Accuracy, sensitivity, and specificity of the model were 82%, 75.6%, and 74.9%, respectively.

For recurrence > 5 years, stage 2 cancer (OR 1.67, 95% CI = 1.31–2.14) and radiotherapy+mastectomy (OR 2.45, 95% CI = 1.81–3.32) were significant predictors; furthermore, relative to mastectomy without radiotherapy (as reference for comparison), quadranectomy without radiotherapy had a noticeably higher odds ratio compared to quadranectomy with radiotherapy for recurrence > 5 years (OR 7.62, 95% CI = 1.52–38.15 vs. OR 1.75, 95% CI = 1.32–2.32). Accuracy, sensitivity, and specificity of the model were 71%, 78.8%, and 55.8%, respectively.

**Conclusion:**

For the first time, we constructed models for estimating recurrence based on timing of recurrence which are among the most applicable models with excellent accuracy (> 80%).

**Electronic supplementary material:**

The online version of this article (10.1186/s12957-018-1489-0) contains supplementary material, which is available to authorized users.

## Background

Breast cancer (BC) is the most common cancer among women and is considered to be the second cause of death among all cancer-related deaths in women [1].

The main treatment of BC is surgery. Recurrence poses a major concern after surgical treatment of BC and is associated with a great increase in BC-related death, which usually occurs during the first 5 years of diagnosis [2, 3].

To date, multiple models have been introduced for predicting BC prognosis, which have mainly focused on survival [4, 5]. These models have aided in developing guidelines and managing BC patients. However, these models have utilized limited variables regarding patients’ clinical characteristics and some have used machine learning algorithms which have difficult clinical interpretations [6, 7].

Considering that most predictors of recurrence (clinicopathological features and tumor specific characteristics) are highly correlated, we aimed to develop a comprehensive model to predict recurrence which would preclude associational factors. In addition, considering that BC recurrence during early stages and late stages of the disease course significantly affects patients’ quality of life, we hypothesized that predictors of recurrence may differ for early recurrence and late recurrence. Thus, in order to answer the question whether or not predictors of early recurrence (defined as earlier than 5 years) are different from those of late recurrence (later than 5 years), we further developed two other models based on time of recurrence using advanced statistical modeling.

## Methods

### Study settings and patient selection

This study is part of an ongoing BC registry termed the Shiraz Breast Cancer Registry (SBCR), which has started its patient registration program since 2005. The breast clinic is located in Motahhari Medical Clinic, Shiraz, Iran. Patients are referred to the breast clinic from multiple medical health centers within the city and from other provinces (mostly those from Southern Iran). Currently, the registry includes more than 6000 registered patients with BC and data on more than 200 variables on patient and clinical characteristics have been documented for each individual within the registry.

Participants were selected from the SBCR, and all individuals diagnosed with BC since 1995 have been included in the current study. All male cases of BC were excluded.

Patients were categorized into three groups according to their recurrence time: those who presented with recurrence during the first 5 years of their initial diagnosis of BC, those who had recurrence after 5 years of their initial diagnosis, and those who did not present with any recurrence more than 10 years from diagnosis.

### Variable selection

More than 35 variables on baseline characteristics, socioeconomic determinants, obstetrics and gynecological history, family history, history of other diseases and other tumors, BC specifics including side of involvement, type of BC, treatment specifics, staging and grading of tumor, and histopathological features were considered and compared between the groups.

Education level was defined as illiterate, high school or less, and college education.

Job of individuals was classified as stay at home, retired, governmental job, and self-employment.

Regarding sports activity patients either said yes or no to having sport activities. Regularity of sport activity was also questioned (either regular or irregular sports activity).

Axillary management was classified as either sentinel lymph node biopsy (SLNB), axillary lymph node dissection (AND), both, or none.

Breast surgery was classified as either mastectomy or breast conserving surgery (BCS).

Histopathological in situ component and tumor necrosis were either existing or not.

### Statistical analysis

All 1273 individuals were included in the final model for estimating patient recurrence. End point was considered metastasis (local, regional, and distant). Primary outcome was considered from time of diagnosis to confirmed recurrence. Initially, individuals were classified as either with recurrence or without recurrence (more than 10 years) and compared. After which, in order to clarify the differences between those with early recurrence and those with late recurrence, we categorized patients into three groups based on their timing of recurrence as those with < 5 years recurrence, > 5-year recurrence, and those without recurrence of more than 10 years. For constructing a model for assessing estimates of recurrence in the population, as we had a large sample size from the BC registry, an efficient algorithm called group LASSO was used to simultaneously perform variable selection and to estimate risk factors in a logistic regression model. In situations that variables present in several levels and can be expressed through a group of dummy variables, group LASSO is suggested. Group LASSO also has excellent properties in terms of both variable importance and prediction and avoids over-shrinking large coefficients. By placing constrain on the absolute value of regression coefficients, the penalized function shrinks many of the coefficients. Furthermore, by deleting additional and redundant variables and creating a brief bias in the models, the group LASSO method controls existing multi-collinearity and is excellent in the settings of high number of variables [8]. Ten-fold cross validation was used to estimate amounts of penalty and bootstrap with 1000 replications was applied to calculate standard error of coefficients. To investigate prediction accuracy of the proposed model in classification of patients with and without recurrence, receiver operator characteristic (ROC) curve analysis was performed and optimal cut off point for obtained probability of BC recurrence was reported; in addition, area under the curve (AUC), sensitivity, and specificity of the obtained cut-off point were also reported. Statistical analysis was performed using SPSS 18.0 and grpreg package in R 3.3.1 software. Considering the main research question, we further classified patients into two groups of those with early recurrence (< 5 years) and late recurrence (> 5 years) and constructed models to predict recurrence in each of these groups, separately.

For evaluation of radiotherapy, considering that indications of radiotherapy differ according to type of breast surgery (either mastectomy or BCS), individuals were first categorized based on type of surgery and radiotherapy was then evaluated in each groups, separately.

Statistical tests were two-sided, and a *p* value of less than 0.05 was considered statistically significant.

## Results

Patients’ baseline characteristics and comparison of individuals with recurrence and those without recurrence are shown in Table 1.

**Table 1:** Baseline and clinicopathological characteristics of individuals with and without recurrence^*^

Variables	Recurrence		Overall (*n* = 1273)	*p* value
	Yes (*n* = 712)	No (*n* = 561)		
Age, years	55.29 ± 11.52	60.26 ± 9.86	57.49 ± 11.10	< 0.001
Number of pregnancies	1.31 ± 2.28	1.92 ± 2.43	1.58 ± 2.37	< 0.001
Number of abortions	0.22 ± 0.68	0.28 ± 0.66	0.22 ± 0.68	0.126
Number of children	1.26 ± 2.18	1.83 ± 2.32	1.51 ± 2.26	< 0.001
Breast feeding duration, months	20.23 ± 38.84	29.43 ± 47.19	24.28 ± 42.95	< 0.001
Tumor size, cm	3.15 ± 1.69	2.87 ± 1.53	3.02 ± 1.63	< 0.001
Duration of sports activity, h/week	0.55 ± 2.15	0.79 ± 1.87	0.66 ± 2.03	0.042
Breast side involvement, no. (%)				
Left	387 (54.4)	285 (50.8)	662 (52)	0.208
Right	325 (45.6)	276 (49.2)	601 (47)	
Hormone replacement therapy, no. (%)				
Yes	5 (0.7)	5 (0.9)	10 (0.8)	0.704
No	707 (99.3)	556 (99.1)	1263 (99.2)	
Diabetes, no. (%)				
Yes	55 (7.7)	75 (13.4)	130 (10.2)	0.001
No	657 (92.3)	486 (86.6)	1143 (89.8)	
History of breast disease, no. (%)				
Yes	14 (2)	9 (1.6)	23 (1.8)	0.630
No	698 (98)	552 (98.4)	1250 (98.2)	
History of breast operation, no. (%)				
Yes	5 (0.7)	7 (1.2)	12 (0.9)	0.317
No	707 (99.3)	554 (98.8)	1261 (99.1)	
Family Hx of breast cancer, no. (%)				
Yes	73 (10.3)	76 (13.5)	149 (11.7)	0.69
No	639 (89.7)	485 (86.5)	1124 (88.3)	
Family Hx of other cancers, no. (%)				
Yes	121 (17)	124 (22.1)	245 (19.2)	0.022
No	591 (83)	437 (77.9)	1028 (80.8)	
Smoking, no. (%)				
Yes	5 (0.7)	2 (0.4)	7 (0.5)	0.408
No	707 (99.3)	559 (99.6)	1266 (99.5)	
Waterpipe use, no. (%)				
Yes	26 (3.7)	26 (4.6)	52 (4.1)	0.379
No	686 (96.3)	535 (95.4)	1221 (95.9)	
Sports activity, no. (%)				
Yes	87 (12.2)	104 (18.5)	191 (15)	0.002
No	626 (87.8)	457 (81.5)	1082 (85)	
Regular sports activity, no. (%)				
Yes	32 (4.5)	47 (8.4)	79 (6.2)	0.004
No	680 (95.5)	514 (91.6)	1194 (93.8)	
Lymph node management, no. (%)				
AND	578 (81.2)	506 (90.2)	1084 (85.2)	< 0.001
SLNB	52 (7.3)	13 (2.3)	65 (5.1)	
Both	40 (5.6)	3 (0.5)	43 (3.4)	
None	42 (5.9)	39 (7)	81 (6.4)	
In situ component, no. (%)				
Yes	365 (51.3)	191 (34)	556 (43.7)	< 0.001
No	347 (48.7)	370 (66)	717 (56.3)	
Tumor necrosis, no. (%)				
Yes	297 (41.7)	132 (23.5)	429 (33.7)	< 0.001
No	415 (58.3)	429 (76.5)	844 (66.3)	
Chemotherapy before surgery, no. (%)				
Yes	60 (8.4)	49 (8.7)	109 (8.6)	0.846
No	652 (91.6)	512 (91.3)	1164 (91.4)	
Chemotherapy after surgery, no. (%)				
Yes	695 (97.6)	541 (96.4)	1236 (97.1)	0.214
No	17 (2.4)	20 (3.6)	37 (2.9)	
Radiotherapy, no. (%)				
Mastectomy with radiotherapy	431 (60.5)	265 (47.2)	696 (54.7)	< 0.001
Mastectomy without radiotherapy	46 (6.5)	123 (21.9)	169 (13.3)	
BCS with radiotherapy	221 (31)	172 (30.7)	393 (30.9)	
BCS without radiotherapy	14 (2)	1 (0.2)	15 (1.2)	
Hormone therapy, no. (%)				
Yes	607 (85.3)	503 (89.7)	1110 (87.2)	0.019
No	105 (14.7)	58 (10.3)	163 (12.8)	
Stage, no. (%)				
0	84 (11.8)	116 (20.7)	200 (15.7)	< 0.001
1	248 (34.8)	302 (53.8)	550 (43.2)	
2	314 (44.1)	126 (22.5)	440 (34.6)	
3 and 4	66 (9.3)	17 (3)	83 (6.5)	
Histological grade, no. (%)				
1, 2	585 (82.2)	511 (91.1)	1096 (86.1)	< 0.001
3	127 (17.8)	50 (8.9)	177 (13.9)	

*SLNB* sentinel lymph node biopsy; *AND* axillary node dissection, *BCS* breast conserving surgery (quadrantectomy)

^*^All plus minus values are means ± standard deviations unless stated otherwise

We further compared those with early and late recurrence and compared them to those without recurrence. Individuals without recurrence more than 10 years were significantly older than those with recurrence > 5 years and recurrence of < 5 years (60.27 ± 9.87, 59.18 ± 9.67, and 54.25 ± 11.76 years old, respectively; *p* < 0.001) had more pregnancies (1.92 ± 2.43, 1.39 ± 2.25, and 1.29 ± 2.29, respectively; *p* < 0.001), more children (1.83 ± 2.32, 1.35 ± 2.20, and 1.24 ± 2.176, respectively; *p* < 0.001), higher rates of retired individuals (18.6%, 13.6%, and 8.5%, respectively, *p* = 0.038), higher rates of diabetes (13.4%, 13.2%, and 6.2%, respectively; *p* < 0.001), higher rates of sports activity (18.5%, 12.6%, and 12.1%, respectively, *p* = 0.007), higher rates of scheduled sports activity (8.4%, 5.3%, and 4.3%, respectively, *p* = 0.015), lower rates of radiotherapy (78.8%, 88.3%, and 87.4%, respectively, *p* < 0.001), and lower stages of BC (21.3%, 16.9%, and 11.9% for stage 1, respectively, *p* < 0.001). Those with recurrence of < 5 years had higher rates of left-sided breast involvement compared to those with recurrence > 5 years and those with no recurrence after 10 years (56%, 45.6%, and 50.5%, respectively, *p* = 0.040), and higher rates of tumor necrosis (58.3%, 39.8%, and 45.1%, respectively, *p* < 0.001).

Those with recurrence of > 5 years had higher rates of other types of cancer in family members compared to the < 5 years recurrence group and those without recurrence > 10 years (25.8%, 14.6%, and 22.1%, respectively, *p* < 0.001), and higher rates of hormone therapy (88.6%, 76.7%, and 86.3%, respectively, *p* < 0.001).

The three groups were also significantly different regarding invasion status (*p* < 0.001) and pathological grade (*p* < 0.001) (Table 2).

**Table 2:** Comparison of clinicopathological characteristics of breast cancer according to timing of recurrence as early and late recurrence^*^

Variables	Rec < 5 years (*n* = 561)	Rec > 5 years (*n* = 151)	No rec > 10 years (*n* = 561)	*p* value
Age, years	54.25 ± 11.76	59.18 ± 9.67	60.26 ± 9.86	< 0.001
Number of pregnancies	1.29 ± 2.29	1.39 ± 2.25	1.92 ± 2.43	< 0.001
Number of abortions	0.22 ± 0.703	0.20 ± 0.62	0.28 ± 0.66	0.287
Number of children	1.24 ± 2.176	1.35 ± 2.20	1.83 ± 2.32	< 0.001
Breast feeding duration, months	20.12 ± 39.71	20.64 ± 35.56	29.43 ± 47.19	0.001
Tumor size, cm	3.21 ± 1.80	0.50 ± 1.46	2.87 ± 1.53	< 0.001
Duration of sports activity, h/week	0.57 ± 2.30	0.05 ± 1.46	0.79 ± 1.87	< 0.001
Breast side involvement, no. (%)				
Left	311 (56)	68 (45.6)	285 (50.8)	0.040
Right	244 (44)	81 (54.4)	276 (49.2)	
Hormone replacement therapy, no. (%)				
Yes	3 (1.4)	2 (3.3)	5 (0.9)	0.611
No	204 (98.6)	58 (96.7)	556 (99.1)	
Diabetes, no. (%)				
Yes	35 (6.2)	20 (13.2)	75 (13.4)	< 0.001
No	526 (93.8)	131 (86.8)	486 (86.6)	
History of breast disease, no. (%)				
Yes	13 (2.3)	1 (0.7)	9 (1.6)	0.355
No	548 (97.7)	150 (99.3)	552 (98.4)	
History of breast operation, no. (%)				
Yes	2 (0.4)	3 (2)	7 (1.2)	0.112
No	559 (99.6)	148 (98)	554 (98.8)	
Family Hx of breast cancer, no. (%)				
Yes	57 (10.2)	16 (10.6)	76 (13.5)	0.190
No	504 (89.8)	135 (89.4)	485 (86.5)	
Family Hx of other cancer, no. (%)				
Yes	82 (14.6)	39 (25.8)	124 (22.1)	0.001
No	479 (85.4)	112 (74.2)	437 (77.9)	
Smoking, no. (%)				
Yes	5 (0.9)	0	2 (0.4)	0.299
No	556 (99.1)	151 (100)	559 (99.6)	
Waterpipe use, no. (%)				
Yes	22 (3.9)	4 (2.6)	26 (4.6)	0.531
No	539 (96.1)	147 (97.4)	535 (95.4)	
Sports activity, no. (%)				
Yes	68 (12.1)	19 (12.6)	104 (18.5)	0.007
No	493 (87.9)	132 (87.4)	457 (81.5)	
Regular sports activity, no. (%)				
Yes	24 (4.3)	8 (5.3)	47 (8.4)	0.015
No	537 (95.7)	143 (94.7)	514 (91.6)	
Lymph node management, no. (%)				
SLNB	48 (8.6)	6 (4)	506 (90.2)	< 0.001
AND	438 (78.1)	126 (83.4)	13 (2.3)	
Both	34 (6.1)	3 (2)	3 (0.5)	
None	41 (7.3)	16 (10.6)	39 (7)	
In situ component, no. (%)				
Yes	310 (69.7)	55 (69.6)	191 (34)	0.578
No	135 (30.3)	24 (30.4)	370 (66)	
Tumor necrosis, no. (%)				
Yes	264 (58.3)	33 (39.8)	132 (23.5)	< 0.001
No	189 (41.7)	50 (60.2)	429 (76.5)	
Chemotherapy before surgery, no. (%)				
Yes	53 (9.4)	7 (4.6)	49 (8.7)	0.169
No	508 (90.6)	144 (95.4)	512 (91.3)	
Chemotherapy after surgery, no. (%)				
Yes	547 (97.5)	148 (98)	541 (96.4)	0.438
No	14 (2.5)	3 (2)	20 (3.6)	
Radiotherapy, no. (%)				
Mastectomy with radiotherapy	166 (29.6)	139 (92.1)	265 (47.2)	< 0.001
Mastectomy without radiotherapy	2 (0.4)	0	123 (21.9)	
BCS with radiotherapy	380 (67.7)	10 (6.6)	172 (30.7)	
BCS without radiotherapy	13 (2.3)	2 (1.3)	1 (0.2)	
Hormone therapy, no. (%)				
Yes	303 (76.7)	101 (88.6)	365 (86.3)	< 0.001
No	92 (23.3)	13 (11.4)	58 (13.7)	
Stage, no. (%)				
0	129 (23)	12 (7.9)	116 (20.7)	< 0.001
1	278 (49.6)	84 (55.6)	302 (53.8)	
2	134 (23.9)	48 (31.8)	126 (22.5)	
3 and 4	20 (3.6)	7 (4.6)	17 (3)	
Grade, no. (%)				
1, 2	448 (79.9)	137 (90.7)	511 (91.1)	< 0.001
3	113 (13.9)	14 (9.3)	50 (8.9)	

*Rec* recurrence; *SLNB* sentinel lymph node biopsy; *AND* axillary node dissection; *BCS* breast conserving surgery

^*^All plus minus values are means ± standard deviations unless stated otherwise

In the LASSO regression model, sports activity (OR 0.69; 95% CI = 0.53–0.91), number of lymph nodes (LN) dissected in SLNB and AND (OR 0.97; 95% CI = 0.96–0.98), and higher age (OR 0.97; 95% CI = 0.96–0.97) were associated with later recurrence, respectively. Moreover, number of invasive LNs in dissection (OR 1.08; 95% CI = 1.06–1.10), in situ component (OR 1.14; 95% CI = 1.14–1.50), tumor necrosis (OR 1.59; 95% CI = 1.35–1.86), breast diseases (OR 1.79; 95% CI = 1.11–2.88), grade 3 (compared to grade 1 and 2) (OR 1.49; 95% CI = 1.22–1.82), smoking (OR 3.76; 95% CI = 1.54–9.16), SLNB (OR 2.62; 95% CI = 1.86–3.68), both SLNB and AND (OR 3.40; 95% CI = 1.55–7.46) (considering AND as base for comparison), radiotherapy with mastectomy (compared to mastectomy without radiotherapy) (OR 2.97; 95% CI = 2.39–3.69), and higher stage of BC [stage 2 (OR 2.43; 95% CI = 1.99–2.97) and stages 3 and 4 (OR 3.53; 95% CI = 2.46–4.56)] were predictors of recurrence (Table 3).

**Table 3:** Risk factor assessment for overall recurrence based on group LASSO analysis

Variables	Odds ratio	95% confidence interval
Number of pregnancies	0.95	0.87–1.04
Number of abortion	1.00	0.91–1.09
Number of children	1.00	0.93–1.08
Breast feeding duration	1.00	1.00–1.00
Right-sided breast involvement	0.90	0.80–1.01
Hormone replacement therapy	1.00	0.55–1.82
Diabetes	0.87	0.72–1.07
History of breast operation	0.80	0.44–1.44
Family history of breast cancer	0.93	0.79–1.10
Family history of other cancers	1.00	0.87–1.15
Waterpipe use	1.00	0.74–1.35
Regular sports activity†	0.79	0.59–1.06
Sports duration	1.00	0.96–1.05
Tumor size	1.00	0.97–1.03
Chemotherapy before surgery	1.00	0.84–1.19
Chemotherapy after surgery	1.00	0.73–1.37
Hormone therapy	0.93	0.78–1.10
Sports activity	0.69^*^	0.53–0.91
Age	0.97^*^	0.96-0.97
Number of lymph nodes dissected	0.97^*^	0.96–0.98
Number of invasive lymph nodes in dissection	1.08^*^	10.6–1.10
In situ component	1.31^*^	1.14–1.50
Grade 3‡	1.49^*^	1.22–1.82
Tumor necrosis	1.59^*^	1.35–1.86
Breast disease	1.79^*^	1.11–2.88
Smoking	3.76^*^	1.54-9.16
Axillary management§		
Sentinel lymph node biopsy	2.62^*^	1.86–3.68
Both	5.48^*^	3.28-9.16
No axillary management	0.91	0.69–1.20
Radiotherapy||		
Yes with mastectomy	2.97^*^	2.39–3.69
Yes with breast conserving surgery	2.34^*^	1.89–2.90
No with breast conserving surgery	13.35^*^	4.99–35.67
Staging of breast cancer¶		
Stage 1	1.13	0.94–1.35
Stage 2	2.43^*^	1.99–2.97
Stages 3 and 4	3.35^*^	2.46–4.56

^*^Shows statistical significance (*p* < 0.05)

†Irregular sports activity was considered base for comparison

‡Grades 1 and 2 were considered base for comparison

§Having axillary lymph node dissection was considered base for comparison

||Having mastectomy without radiotherapy was considered base for comparison

¶Stage zero was considered base for comparison

**Fig. 1:**
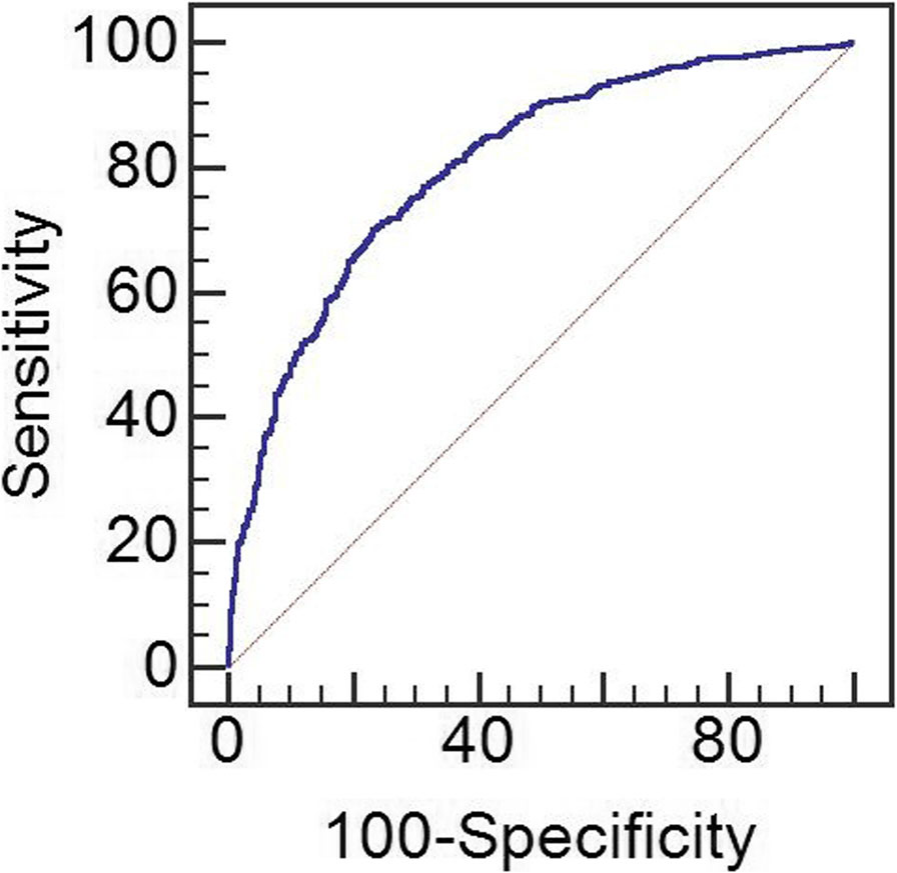
ROC curve for model predicting overall recurrence

Using coefficients, probability of BC recurrence was calculated for each patient. Cut-off point was determined as *p* = 0.566 in ROC analysis and accuracy of the proposed model was equal to 80% (95% CI = 78.2–82.6%). Furthermore, sensitivity and specificity for group LASSO was 70.1% and 76.8%, respectively. Tuning parameter for this model was 0.006 (Fig. 1).

When stratified according to timing of recurrence, our models showed that for recurrence < 5 years, age (OR 0.96, 95% CI = 0.95–0.97), number of pregnancies (OR 0.94, 95% CI = 0.89–0.99), family history of other cancers (OR 0.73, 95% CI = 0.60–0.89), hormone therapy (OR 0.76, 95% CI = 0.61–0.96), total number of dissected LN (OR 0.98, 95% CI = 0.97–0.99), right-sided BC (OR 0.87, 95% CI = 0.77–0.99), diabetes (OR 0.77, 95% CI = 0.60–0.98), and history of breast operations (OR 0.38, 95% CI = 0.17–0.88) were protective against recurrence. However, smoking (OR 5.72, 95% CI = 2.11–15.55), history of previous breast disease (OR 3.32, 95% CI = 1.92–5.76), in situ component (OR 1.58, 95% CI = 1.35–1.84), tumor necrosis (OR 1.87, 95% CI = 1.57–2.22), SLNB (OR 2.90, 95% CI = 2.05–4.11) and concomitant SLNB and AND (OR 3.50, 95% CI = 2.26–5.42), grade 3 (OR 1.79, 95% CI = 1.46–2.21), stage 2 (OR 2.71, 95% CI = 2.18–3.35), stages 3 and 4 (OR 5.01, 95% CI = 3.52–7.13) and mastectomy with radiotherapy (OR 2.97, 95% CI = 2.39–3.68) were predictors of worse < 5-year recurrence. Moreover, relative to mastectomy without radiotherapy (as reference for comparison), quadranectomy without radiotherapy had a noticeably higher odds ratio compared to quadranectomy with radiotherapy for recurrence < 5 years (OR 17.58, 95% CI = 6.70–46.10 vs. OR 2.50, 95% CI = 2–3.12) (Table 4).

**Table 4:** Comparison of estimates of recurrence between those with early and late recurrence using group LASSO analysis

Variables	Recurrence			
	< 5 years		> 5 years	
	Odds ratio	95% CI	Odds ratio	95% CI
Age	0.96^*^	0.95–0.97	1.00	0.99–1.01
Number of pregnancies	0.94^*^	0.89–0.99	0.96	0.91–1.00
Family history of other cancers	0.73^*^	0.60–0.89	1.00	0.85–1.17
Hormone therapy	0.76^*^	0.61–0.96	1.00	0.83–1.20
Total lymph nodes dissected	0.98^*^	0.97–0.99	1.00	0.99–1.01
Right-sided breast involvement	0.87^*^	0.77–0.99	1.00	0.89–1.13
Diabetes	0.77^*^	0.60-0.98	1.00	0.83–1.21
History of breast operation	0.38^*^	0.17–0.88	1.00	0.65–1.53
Sports activity	0.79	0.60–1.05	1.00	0.68–1.47
Regular sports activity†	0.80	0.57–1.13	1.00	0.80–1.24
Sports duration	1.00	0.95–1.05	1.00	0.81–1.24
Number of abortions	1.04	0.95–1.14	1.00	0.87–1.14
Number of children	1.00	0.96–1.04	1.00	0.98–1.02
Duration of breast feeding	1.00	1.00–1.00	1.00	1.00–1.00
Hormone replacement therapy	1.04	0.41–2.63	1.00	0.63–1.60
Family history of breast cancer	1.00	0.86–1.17	1.00	0.53–1.87
Waterpipe use	1.00	0.72–1.39	1.00	0.96–1.04
Smoking	5.72^*^	2.11-15.55	1.32	1.00–1.73
History of breast disease	3.32^*^	1.92–5.76	1.00	0.56–1.79
In situ component	1.58^*^	1.35–1.84	1.00	0.91–1.10
Tumor necrosis	1.87^*^	1.57–2.22	1.00	0.85–1.17
Axillary management‡				
Sentinel lymph node biopsy	2.90^*^	2.05–4.11	1.00	0.67–1.48
both	3.50^*^	2.26-5.42	1.00	0.63–1.58
No axillary management	0.81	0.63–1.05	1.01	0.80–1.29
Grade 3§	1.79^*^	1.46–2.21	1.00	0.82–1.22
Staging of breast cancer||				
Stage 1	1.16	0.95–1.42	0.97	0.81–1.16
Stage 2	2.71^*^	2.18–3.35	1.67^*^	1.31-2.14
Stages 3 and 4	5.01^*^	3.52–7.13	1.31	0.83–2.05
Radiotherapy¶				
Yes with mastectomy	2.97^*^	2.39–3.68	2.45^*^	1.81-3.32
Yes with breast conserving surgery	2.50^*^	2.00–3.12	1.75^*^	1.32-2.32
No with breast conserving surgery	17.58^*^	6.70–46.10	7.62^*^	1.52-38.15
Invasive LN in dissection	1.00	0.99–1.01	1.00	0.98–1.01
Chemotherapy before surgery	1.00	0.83–1.20	0.76	0.54–1.07
Chemotherapy after surgery	1.00	0.66–1.50	1.00	0.72–1.39
Tumor size	1.02	0.98–1.07	0.97	0.93–1.01

^*^Shows statistical significance (*p* < 0.05)

†Irregular sports activity was considered base for comparison

‡Having axillary lymph node dissection was considered base for comparison

§Grades 1 and 2 were considered base for comparison

||Stage zero was considered base for comparison

¶Having mastectomy without radiotherapy was considered base for comparison

**Fig. 2:**
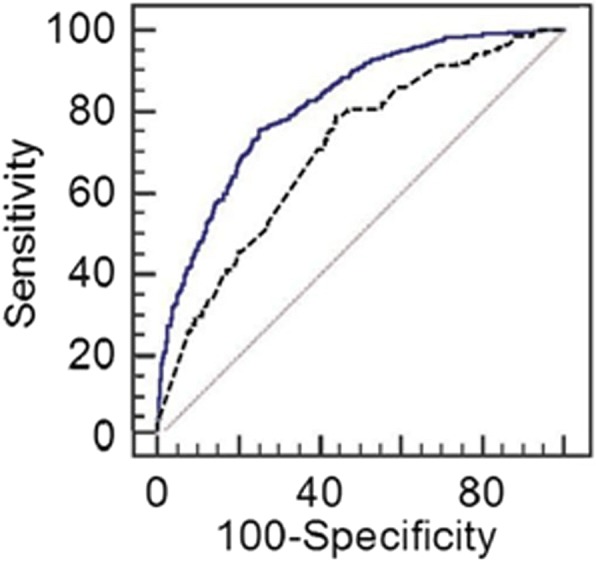
ROC curve for model predicting recurrence less than 5 years (upper line) and for model predicting recurrence more than 5 years (lower line)

Cut-off point for this model (< 5-year recurrence) was determined as *p* = 0.495 in ROC analysis and accuracy of the proposed model was equal to 82% (95% CI = 80–84%). Sensitivity and specificity for group LASSO was 75.6% and 74.9%, respectively. Tuning parameter for this model was 0.006 (Fig. 2).

For recurrence of > 5 years, only stage 2 cancer (OR 1.67, 95% CI = 1.31–2.14) and radiotherapy in mastectomy (OR 2.45, 95% CI = 1.81–3.32) were predictors of worse recurrence; furthermore, relative to mastectomy without radiotherapy (as reference for comparison), quadranectomy without radiotherapy had a noticeably higher odds ratio compared to quadrantectomy with radiotherapy for recurrence > 5 years (OR 7.62, 95% CI = 1.52–38.15 vs. OR 1.75, 95% CI = 1.32–2.32).

Cut-off point for this model (> 5-year recurrence) was determined as *p* = 0.206 in ROC analysis and accuracy of the proposed model was equal to 71% (95% CI = 67–74%). Sensitivity and specificity for group LASSO were 78.8% and 55.8%, respectively. Tuning parameter for this model was 0.007 (Fig. 2).

The three final models are provided in Additional file 1, which can be utilized to estimate recurrence time based on our selected variables; furthermore, the models will also be available at the breast clinic website at www.bdrc.sums.ac.ir.

## Discussion

In here, we aimed to introduce models to predict recurrence in a large sample of individuals during a period of 20 years from 1995 to 2016. We further defined a model to predict recurrence among those with early recurrence and late recurrence and compared estimates.

In our final model which included more than 50 variables on different aspects of BC and patient baseline characteristics, we found that aside to more common and previously known risk factors like clinical stage, and pathological grade, factors like sports activity, higher age, number of LNs dissected in SLNB and AND, and radiotherapy in BCS were protective against recurrence, on the other hand, in situ component in pathology, tumor necrosis, having other breast diseases, smoking, LN management including SLNB, and simultaneous SLNB and AND (considering AND as base for comparison), number of invasive LNs after dissection, and radiotherapy after mastectomy were associated with earlier recurrence.

When we stratified our models based on early and late recurrence, we found that for early recurrence (< 5 years), in addition to factors that were significant for overall recurrence, number of pregnancies, family history of other cancers, hormone therapy, right-sided BC, diabetes, and history of breast operations were predictors of better outcome. Furthermore, only stage 2 BC and radiotherapy were significant predictors in late recurrence (> 5 years).

Recently, Wu et al. [9] introduced a model for estimating 5-year recurrence in a population of 4505 women. In their final model, they found age of less than 54 years old, alcohol consumption and adjuvant therapy to be protective, African American ethnicity, nuclear grade 3, tumor size, number of positive nodes, and lymphovascular invasion to be malignant predictors of 5-year recurrence. They introduced one of the most comprehensive models for estimating 5-year recurrence using both epidemiological data and BC specific data and by using a Cox analysis approach. Similar to the mentioned study, we had one of the most comprehensive models for predicting BC recurrence in two phases of early recurrence and late recurrence. Furthermore, as we included a wide range of data from our BC registry, we introduced a more comprehensive model including baseline characteristics, socioeconomic determinants, obstetrics and gynecological data, pathological data, and personal habits like smoking and sports activity. In our results, we also found a number of positive nodes and grade to be predictors of worse recurrence.

Considering the clinical value of timing of recurrence, we introduced two models according to time of recurrence as early and late recurrence. Accordingly, our models showed that only radiotherapy and stage of cancer remained to be significant in recurrence of > 5 years. This is an important clinical finding as it aids significantly in the understanding of late recurrence in BC patients.

In a smaller study in 2016 [10], those with early (< 5 years) and those with late recurrence (> 5 years) were compared regarding clinical characteristics. They found that these two groups differed regarding tumor size, number of positive nodes, grade, *ER* and *PR* receptors and *HER2*, and adjuvant therapy. In their multivariate regression models, they found tumor size, *ER* receptor and *HER2* to be associated with worse > 5-year recurrence and grade 2 BC to be associated with better late recurrence. They used regression modelling to estimate predictors of late recurrence in a population of 300 women, and their study did not provide an overview of differences between those who present with early and those who present with late recurrence as they only had limited set of participants and variables. Another study in 2016 [11] evaluated factors associated with BC recurrence after BCS and found premenopausal state, *ER* expression, and hormone therapy to be factors associated with recurrence. Similarly, we also found hormone therapy to be significant in our < 5-year recurrence model.

Our study presents a novel assessment of BC recurrence, and accordingly, we found some interesting results regarding determinants of BC recurrence using advanced statistical modeling.

Among the most interesting findings was the association between sports activity and recurrence, although sports activity was measured in a subjective manner and patients were asked regarding their daily routine and physical activity, sports activity presented as highly protective in BC recurrence. Studies on recurrence and physical activity in the settings of a large sample with long-term follow-up were mainly missing up to 2006 according to a meta-analysis by McNeely [12] in 2006 who evaluated the relationship between exercise and BC. To date, most studies have mostly focused on physical activity and BC outcomes as a whole; however, more recently two studies evaluated the association between exercise and BC recurrence, one was conducted in Germany and another in a Canadian registry. A meta-analysis in 2015 [13] found that using data from the two mentioned studies, exercise showed a protective role against recurrence with an odds ratio very similar to that of our study (OR 0.72; 95% CI = 0.56–0.91). Although the exact mechanism by which exercise decreases recurrence rates still remains unknown, studies have shown exercise to improve quality of life in BC patients [12], and others have also attributed this to changes in adipose tissue and skeletal muscle [14].

Regarding pathology-related parameters, in situ component and tumor necrosis were associated with worse recurrence.

We found those who had both SLNB and AND were at higher risk of recurrence when compared to those who had isolated SLNB or AND alone, respectively.

As SLNB has recently been added as a treatment modality to replace isolate AND [15], furthermore considering isolated SLNB has recently been accepted and applied in our center and in literature, and our study included patients from 1995 which is before the introduction of SLNB to replace AND, some patients that had axillary dissection may have been node negative in the past (a mixture of both patients with good and bad prognosis). This may have been among the reasons for the higher recorded recurrence rate associated with SLNB (compared to AND), thus, judgment on the matter should be done with caution. Those who had both axillary management modalities had definitive positive LNs and consequently had worse prognosis. However, all these are mainly considered for locoregional recurrence, and distant metastasis presents more complicated phenomena and may not be easily explained. Although, AND is not considered among patient without palpable masses or signs of metastasis in sonography evaluation, a review in 2013 [16] found that among individuals without the mentioned conditions, AND improves recurrence rates by 1–3% compared to isolated SLNB, which was similar to our results. In a more recent review by Bromham and colleagues [17] that included RCT’s comparing individuals with no axillary surgery and those with AND, they found that no axillary surgery increased locoregional recurrence by 1.10 to 3.06; however, regarding distant metastasis, they found uncertain results as to whether no surgery increased metastasis rates (HR 1.06; 95% CI = 0.87–1.30). Comparing isolated AND and SLNB showed uncertain results regarding distant metastasis in the mentioned study (HR 0.80; 95% CI = 0.42–1.53).

Among the interesting findings was that number of LNs dissected in AND or SLNB management was associated with better overall recurrence. On the other hand, number of invasive nodes detected in AND and SLNB was associated with worse recurrence. These findings should be considered with caution regarding its clinical application as higher number of dissection will ultimately produce higher complications such as lymphedema.

In our model history of breast diseases presented as a strong risk factor for recurrence, this is a novel concept yet to be described and evaluated.

Those with mastectomy who received radiotherapy had earlier recurrence than those who only had mastectomy without radiotherapy; this is attributable to the more advanced stages of patient who receive concomitant mastectomy and radiotherapy and is expected.

Among other novel findings in our endeavor to find associated factors with recurrence, was the association of smoking with recurrence and the insignificant association of waterpipe use with recurrence. As the use of waterpipes continues to grow worldwide, it has become a global epidemic with recent reports from the middle-east indicating that it has even surpassed cigarette smoking to become the most common form of tobacco used in the region [18]. This is the first study to evaluate waterpipe use in BC recurrence.

We found multiple obstetrics-related variables such as number of pregnancies, number of abortions, number of children, and breast feeding duration to not be significantly associated with overall recurrence, however number of pregnancies, history of breast operation, hormone therapy, right-sided BC, history of previous breast disease demonstrated significance in our < 5-year recurrence model.

We found diabetes to be a good predictor of < 5-year recurrence, which was similar to that reported by Chen et al. [19]. Using the Surveillance, Epidemiology and End-Results (SEER)-Medicare database, they found Metformin use to be associated with a 31% (95% CI 0.53–0.90) decrease in BC recurrence.

Our results indicate that regarding treatment modalities only radiotherapy seems to affect recurrence of > 5 years which renders different results based on type of BC surgery performed for the patient (as either mastectomy or BCS).

This study was not without limitation. As we had limited number of individuals in some of the categories, all variables in our database were not applicable in the final model due to the limited number of data. Taking into consideration that individuals who were recently (less than 10 years from their initial diagnosis) added to our registry may not have had the chance to present signs of recurrence, we considered those without recurrence of more than 10 years from their initial diagnosis of BC and this decreased the size of the comparison groups.

## Conclusion

As the main outcome of our study, we used advanced statistics to construct models based on multiple factors to predict both early and late recurrence. Compared to previous literature which has included limited variables, our models are among the most applicable and comprehensive models for predicting recurrence based on timing of recurrence with excellent accuracy (> 80%).

## Additional file

Additional file 1: Formulas for the prediction of recurrence. (DOCX 15 kb)
