# Supplementary material for: Introducing novel and comprehensive models for predicting recurrence in breast cancer using the group LASSO approach: are estimates of early and late recurrence different?
Source: World J Surg Oncol. 2018 Sep 12;16:185. doi: 10.1186/s12957-018-1489-0 (PMC6136222; doi:10.1186/s12957-018-1489-0)
Supplement: Supplementary file 1 — Formulas for the prediction of recurrence. (DOCX 15 kb) [file 12957_2018_1489_MOESM1_ESM.docx]

**Formulas for the prediction of recurrence**

**Probability of overall recurrence for person i:**

Z_i_=1.15-0.03*x_i1_-0.05*x_i2_-0.10*x_i6_-0.13*x_i8_+0.58*x_i9_-0.22*x_i10_-0.07*x_i11_+1.32*x_i13_-0.37*x_i15_-0.24*x_i16_+0.27*x_i19_+0.46*x_i20_-0.07*x_i23_ +0.96*x_i24_+1.70*x_i25_-0.09*x_i26_-0.03*x_i27_+0.08*x_i28_+0.40*x_i29_+0.12*x_i30_+0.89*x_i31_+1.21*x_i32_ +1.09*x_i33_+2.59*x_i34_+0.85*x_i35_

P_i_=$\frac{exp(Z_{i})}{1+exp(Z_{i})}$

x1: Age

x2: Number of pregnancies

x6: Side of breast involvement (left side = 0, right side = 1)

x9: History of breast disease (no history = 0, positive history = 1)

x10: History of previous breast operation (no history = 0, positive history = 1)

x11: Family history of breast cancer (no history = 0, positive history = 1)

x13: Smoking (no = 0, yes = 1)

x15: Sports activity (no = 0, yes = 1)

x16: Regular sports activity (no = 0, yes = 1)

x19: Insitu component (no = 0, yes = 1)

x20: Tumor necrosis (no = 0, yes = 1)

x23: Hormone therapy (no = 0, yes = 1)

x24: Isolated SLNB (no = 0, yes = 1)

x25: Both SLNB and AND (no = 0, yes = 1)

x26: No axillary management (no = 0, yes = 1)

x27: Total number of lymph nodes dissected

x28: Number of invasive lymph nodes in pathology report

x29: Grade 3 (no = 0, yes = 1)

x30: Stage 1 (no = 0, yes = 1)

x31: Stage 2 (no = 0, yes = 1)

x32: Stage 3 or 4 (no = 0, yes = 1)

x33: Mastectomy + radiotherapy (no = 0, yes = 1)

x34: Breast conserving surgery (quadranectomy) + no radiotherapy (no = 0, yes = 1)

x35: Breast conserving surgery (quadranectomy) + radiotherapy (no = 0, yes = 1)

**Probability of recurrence of less than 5 years for person i:**

Z_i_=1.15-0.04*x_i1_-0.06*x_i2_+0.04*x_i3_-0.14*x_i6_+0.04*x_i7_-0.26*x_i8_+1.2*x_i9_-0.96*x_i10_-0.32*x_i12_-1.74*x_i13_-0.23*x_i15_-0.22*x_i16_+0.02*x_i18_+0.46*x_i19_+0.62*x_i20_-0.27*x_i23_ +1.07*x_i24_+1.25*x_i25_-0.21*x_i26_-0.02*x_i27_+0.58*x_i29_+0.15*x_i30_+1.00*x_i31_+1.61*x_i32_ +1.09*x_i33_+2.87*x_i34_+0.92*x_i35_

P_i_=$\frac{exp(Z_{i})}{1+exp(Z_{i})}$

x1: Age

x2: Number of pregnancies

x3: Number of abortions

x6: Side of breast involvement (left side = 0, right side = 1)

x7: Hormone replacement therapy (no = 0, yes = 1)

x8: Diabetes (no = 0, yes = 1)

x9: History of breast disease (no history = 0, positive history = 1)

x10: History of previous breast operation (no history = 0, positive history = 1)

x12: Family history of other cancers (no history = 0, positive history = 1)

x13: Smoking (no = 0, yes = 1)

x15: Sports activity (no = 0, yes = 1)

x16: Regular sports activity (no = 0, yes = 1)

x18: Tumor size (cm)

x19: Insitu component (no = 0, yes = 1)

x20: Tumor necrosis (no = 0, yes = 1)

x23: Hormone therapy (no = 0, yes = 1)

x24: Isolated SLNB (no = 0, yes = 1)

x25: Both SLNB and AND (no = 0, yes = 1)

x26: No axillary management (no = 0, yes = 1)

x27: Total number of lymph nodes dissected

x29: Grade 3 (no = 0, yes = 1)

x30: Stage 1 (no = 0, yes = 1)

x31: Stage 2 (no = 0, yes = 1)

x32: Stage 3 or 4 (no = 0, yes = 1)

x33: Mastectomy + radiotherapy (no = 0, yes = 1)

x34: Breast conserving surgery (quadranectomy) + no radiotherapy (no = 0, yes = 1)

x35: Breast conserving surgery (quadranectomy) + radiotherapy (no = 0, yes = 1)

**Probability of recurrence of more than five years for person i:**

Z_i_=-2.07-0.04*x_i2_+0.28*x_i13_-0.03*x_i18_-0.27*x_i21_-0.27*x_i23_ +0.01*x_i26_-0.04*x_i30_+0.51*x_i31_+0.27*x_i32_ +0.90*x_i33_+2.03*x_i34_+0.56*x_i35_

P_i_=$\frac{exp(Z_{i})}{1+exp(Z_{i})}$

x2: Number of pregnancies

x13: Smoking (no = 0, yes = 1)

x18: Tumor size (cm)

x21: Chemotherapy before surgery (no = 0, yes = 1)

x23: Hormone therapy (no = 0, yes = 1)

x26: No axillary management (no = 0, yes = 1)

x30: Stage 1 (no = 0, yes = 1)

x31: Stage 2 (no = 0, yes = 1)

x32: Stage 3 or 4 (no = 0, yes = 1)

x33: Mastectomy + radiotherapy (no = 0, yes = 1)

x34: Breast conserving surgery (quadranectomy) + no radiotherapy (no = 0, yes = 1)

x35: Breast conserving surgery (quadranectomy) + radiotherapy (no = 0, yes = 1)
